# Increased Levels of Soluble CD206 Associated with Rapidly Progressive Interstitial Lung Disease in Patients with Dermatomyositis

**DOI:** 10.1155/2020/7948095

**Published:** 2020-10-26

**Authors:** Ya-Wen Shen, Ya-Mei Zhang, Zhen-Guo Huang, Guo-Chun Wang, Qing-Lin Peng

**Affiliations:** ^1^Peking University China-Japan Friendship School of Clinical Medicine, Beijing 100029, China; ^2^Department of Rheumatology, Beijing Key Lab for Immune-Mediated Inflammatory Diseases, China-Japan Friendship Hospital, Beijing 100029, China; ^3^Department of Radiology, China-Japan Friendship Hospital, Beijing 100029, China

## Abstract

**Objective:**

Soluble CD206 (sCD206) is considered a macrophage activation marker, and a previous study proved it as a potential biomarker to predict the severity of anti-melanoma differentiation-associated gene 5- (anti-MDA-5-) positive dermatomyositis- (DM-) associated interstitial lung disease (ILD). To investigate the role of sCD206 in various subtypes of DM, we evaluated the serum level of sCD206 in patients with different myositis-specific autoantibodies besides anti-MDA-5 and clarified its clinical significance.

**Methods:**

Commercial enzyme-linked immunosorbent assay kits were used to detect serum concentrations of sCD206 in 150 patients with DM and 52 healthy controls (HCs). Correlations between sCD206 levels and clinical features, laboratory examinations, and pulmonary function test parameters were analysed.

**Results:**

The median concentrations of serum sCD206 in DM patients were significantly higher than those in HCs (*p* < 0.0001). Furthermore, median sCD206 levels were elevated in patients with ILD (*p* = 0.001), especially in those with rapidly progressive ILD (RP-ILD) (*p* < 0.0001). In addition, sCD206 levels were negatively correlated with the pulmonary function test results, including the percent predicted forced vital capacity (*r* = −0.234, *p* = 0.023), percent predicted forced expiratory volume in one second (*r* = −0.225, *p* = 0.030), and percent predicted carbon monoxide diffusion capacity (*r* = −0.261, *p* = 0.014). Age- and gender-adjusted multivariable analysis showed that sCD206 was an independent prognostic factor for RP-ILD in patients with DM. A longitudinal study showed that sCD206 levels were positively correlated with the physician global assessment visual analog scale scores (*β* = 54.201, *p* = 0.001).

**Conclusion:**

Serum sCD206 levels were significantly increased in patients with DM and significantly associated with RP-ILD, suggesting that sCD206 is an important biological predictor of RP-ILD in patients with DM.

## 1. Introduction

Dermatomyositis (DM) is a group of heterogeneous systemic autoimmune diseases that involves multiple organs such as the muscles, skin, joints, gastrointestinal tract, cardiovascular system, and lungs. Interstitial lung disease (ILD) is considered the most common and severe complication of DM, leading to poor therapeutic effect and prognosis [1–3]. Myositis-specific autoantibodies (MSAs) have been recognised as important biological markers for clinical subtype classification of patients with DM. Among these autoantibodies, the anti-aminoacyl-tRNA synthetase (ARS) antibody and anti-melanoma differentiation-associated gene 5 (MDA-5) antibody are particularly closely associated with ILD [3–8]. Other serum markers such as Krebs von den Lungen-6, ferritin, interleukin 18, and surfactant Protein-D are also believed to be associated with ILD and are used to evaluate the disease activity, therapeutic response, and prognosis [9–14].

As one of the cells in primary barriers for the host to resist pathogens, the macrophage plays key roles in innate and acquired immunity. It is a multifunctional cell with distinct biological functions in different pathophysiological processes such as infection, inflammation, injury repair, cancer, and organ fibrosis according to various local microenvironments [15–19]. CD206 is a type I transmembrane glycoprotein, mainly expressed by the macrophage. It contains the following three extracellular domains, namely, the CR, FNII, and CTLD domains, combining different carbohydrate and protein components. CD206 plays an essential role in eliminating endogenous molecules, promoting antigen presentation, and regulating cell activation and transportation by macrophages [20–24]. It can be cleaved by metalloprotease to produce a soluble form of CD206 [25], and soluble CD206 (sCD206) is considered a macrophage activation marker increasing in various disease states, including sepsis, liver disease, and autoimmunity disease [26–29]. Recently, Horiike et al. reported that serum sCD206 levels were related to the poor prognosis of anti-MDA-5-positive DM-ILD patients [29]. To investigate the role of sCD206 in other subtypes of DM, we evaluated the serum levels of sCD206 in DM patients with different MSAs and explored its clinical significance.

## 2. Materials and Methods

### 2.1. Patients

One hundred and fifty patients with DM admitted to the Department of Rheumatology at the China-Japan Friendship Hospital from March 2005 to November 2016 (including 20 patients with amyopathic dermatomyositis (ADM)), and 52 age- and gender-matched healthy controls (HCs) were retrospectively enrolled in this study. The diagnosis of DM or ADM was based on the criteria of Bohan and Peter [30, 31] or Sontheimer [32]. All of the patients were reevaluated and reclassified following the 2017 European League Against Rheumatism/American College of Rheumatology (EULAR/ACR) classification criteria [33], and finally, 150 patients who fulfilled these classification criteria were enrolled in our study. The exclusion criteria were as follows: (1) age of onset below 18 years and (2) overlap with other connective tissue diseases. Clinical data were retrospectively obtained from hospital medical records. Age of onset was defined as the age at which the first myositis symptom occurred, muscle weakness was defined by manual muscle testing or another objective strength testing, and dysphagia referred to the difficulty in swallowing or objective evidence of abnormal motility of the oesophagus. ILD was diagnosed through high-resolution computed tomography, and rapidly progressive ILD (RP-ILD) was defined as the deterioration of interstitial lesions (including radiologic interstitial worsening accompanied by progressive dyspnoea and other hypoxemia symptoms) occurring within three months after the occurrence of the first respiratory symptoms, according to the “International Consensus Statement of Idiopathic Pulmonary Fibrosis of the American Thoracic Society and the European Respiratory Society” and “Update of the International Multidisciplinary Classification of the Idiopathic Interstitial Pneumonias of the American Thoracic Society and the European Respiratory Society” [34, 35].

This study has been approved by the Research Review Committee and Ethics Review Committee of the China-Japan Friendship Hospital, with registration number 2016-117. Furthermore, written informed consent was obtained from all individuals participating in this study.

### 2.2. Measurement of Serum sCD206 Levels

All serum samples were routinely collected from patients before administering treatments during hospitalization or outpatient clinic visits and stored at -80°C. The concentrations of sCD206 were measured using commercial enzyme-linked immunosorbent assay kits (Human MMR ELISA Kits, RayBiotech, Norcross, GA). The measurement was performed according to the manufacturer's instructions. First, 100 *μ*l of standard solutions or samples was added to each well and incubated for 2.5 hours. Then, after four times of washing, 100 *μ*l of prepared biotin antibodies was added to each well. After an hour of incubation, 100 *μ*l of prepared streptavidin solution was added and incubated for 45 minutes. Afterward, the mixture was washed four times, and 100 *μ*l of TMB one-step substrate reagent was added to each well and incubated for 30 minutes, followed by another four rounds of washing. Finally, after adding 50 *μ*l of stop solution to each well, the samples were read at 450 nm to obtain the OD value of each well. All the incubations were carried out at room temperature, and the concentrations of sCD206 were calculated according to the standard curve.

### 2.3. Detection of MSAs

Commercial immunoblot assays (EUROIMMUN, Luebeck, Germany) were used to detect MSAs such as anti-ARS (including anti-histidyl-tRNA synthetase (Jo-1), anti-threonyl-tRNA synthetase (PL-7), anti-alanyl-tRNA synthetase (PL-12), anti-glycyl-tRNA synthetase (EJ), and anti-isoleucyl-tRNA synthetase (OJ)), anti-MDA-5, anti-transcription intermediary factor 1*γ* (TIF1*γ*), anti-nuclear matrix protein-2 (NXP-2), anti-small ubiquitin-like modifier-1 activating enzyme (SAE), and anti-nucleosome remodelling deacetylase complex (Mi-2), in accordance with the manufacturer's protocol.

### 2.4. Assessment of Disease Activity

A longitudinal study was performed to investigate the correlation of the sCD206 level with disease activity. In this study, a continuous 10 cm visual analog scale (VAS) was used to evaluate the physician global assessment (PGA) of patients with DM, according to core set measures (CSM) for the evaluation of myositis disease activity established by the International Myositis Assessment and Clinical Studies (IMACS) [36]. Twenty DM patients with longitudinal clinical data were enrolled for the follow-up study. Disease activities were assessed during every follow-up visit, and the evaluation of the PGA VAS scores was performed by a physician blinded to the levels of sCD206.

### 2.5. Statistical Analysis

Continuous data were described using mean ± standard deviation (SD) or median (interquartile range (IQR)). The *t*-test was used to compare normal data, while the Mann–Whitney *U* test was used to compare nonnormal data. The Spearman correlation analysis was used to analyse the correlations in the cross-sectional study, and the generalized estimating equation (GEE) was applied to the longitudinal study. In the prediction of RP-ILD, univariate as well as age- and gender-adjusted multivariate logistic regression analyses were performed, and the receiver operating characteristic (ROC) curve and area under the curve (AUC) were used to calculate the best predictive cut-off value. *p* values less than 0.05 were considered statistically significant. Statistical analyses of the data were performed using SPSS version 25.0 and GraphPad Prism version 8.0.

## 3. Results

### 3.1. Clinical Characteristics of Patients with DM

A total of 150 DM patients were included in this study. Among the patients, 108 were women. The mean onset age was 48.87 years, and the median disease duration was 8.5 months. Some (32%) of the patients were treatment naïve; these were patients who did not undergo glucocorticoid and/or immunosuppressive therapy before serum collection. The clinical characteristics, laboratory examinations, and pulmonary function parameters are shown in Table 1.

### 3.2. Serum sCD206 Concentrations in Patients with DM

**Figure 1:**
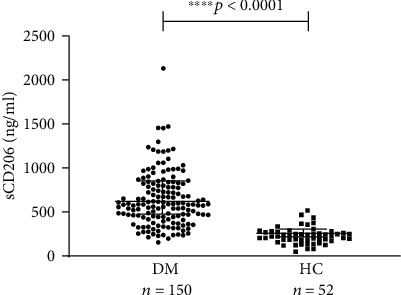
The serum levels of sCD206 in patients with DM and HC samples. The serum levels of sCD206 in DM patients were significantly more elevated than those in HCs. sCD206: soluble CD206; HC: healthy control; DM: dermatomyositis. The error bars represent the interquartile range.

The median level of serum sCD206 in patients with DM was 586.0 ng/ml (440.9-819.8 ng/ml), which was significantly higher than that in the HCs (222.8 ng/ml (183.3-272.4 ng/ml)) (*p* < 0.0001) (Figure 1).

### 3.3. Correlations between sCD206 Levels and Clinical Characteristics in Patients with DM

**Figure 2:**
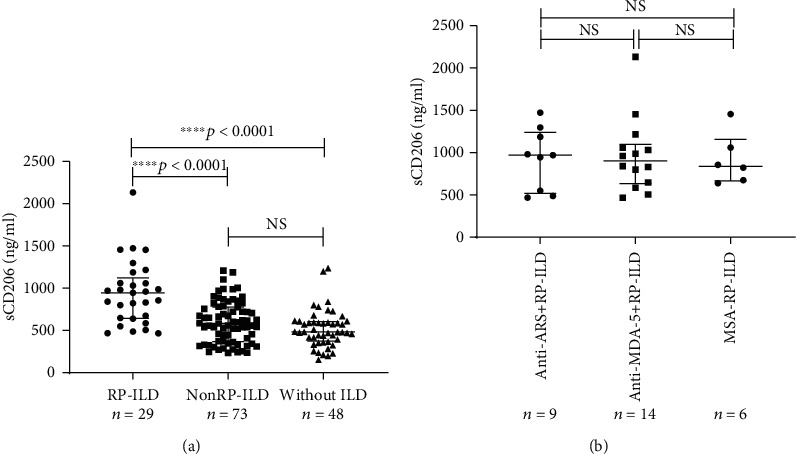
The sCD206 levels in all DM patients and in those with RP-ILD with different MSAs. (a) The serum levels of sCD206 in DM patients with RP-ILD, nonRP-ILD, and those without ILD. (b) The serum levels of sCD206 in DM patients with RP-ILD classified based on different MSAs. sCD206: soluble CD206; DM: dermatomyositis; ILD: interstitial lung disease; RP-ILD: rapidly progressive interstitial lung disease; ARS: aminoacyl-tRNA synthetases; MDA-5: melanoma differentiation-associated gene-5; MSA: myositis-specific autoantibodies. The Kruskal-Wallis *H* test was applied for comparisons between three groups. Error bars represent the interquartile range. NS indicates no significant difference.

We analysed the relationships between sCD206 levels and clinical characteristics of the DM patients and found that the concentrations of sCD206 in patients with ILD (median: 648.2 ng/ml, IQR: 465.2-872.2 ng/ml) were significantly higher than those without ILD (median: 481.5 ng/ml, IQR: 373.3-603.6 ng/ml) (*p* = 0.001). Then, the ILD patients were further divided into two groups: patients with RP-ILD and patients with nonRP-ILD. Interestingly, the serum sCD206 levels in patients with RP-ILD were found to be significantly higher than those with nonRP-ILD (median: 944.6 ng/ml, IQR: 643.1-1122.0 ng/ml vs. median: 582.0 ng/ml, IQR: 367.7-776.7 ng/ml, adjusted *p* < 0.0001). As for patients with nonRP-ILD and patients without ILD, there was no statistical difference in sCD206 levels (median: 582.0 ng/ml, IQR: 367.7-776.7 ng/ml vs. median: 481.5 ng/ml, IQR: 373.3-603.6 ng/ml, adjusted *p* = 0.254) (Figure 2(a)); however, sCD206 concentrations in these two groups of patients were both significantly elevated compared to HCs (median: 582.0 ng/ml, IQR: 367.7-776.7 ng/ml and median: 481.5 ng/ml, IQR: 373.3-603.6 ng/ml vs. median: 222.8 ng/ml, IQR: 183.3-272.4 ng/ml, both *p* < 0.0001). Subsequently, according to the different types of MSAs, RP-ILD patients were divided into the anti-ARS positive, anti-MDA-5 positive, and MSA-negative groups, with no difference of sCD206 levels among these subgroups of patients (*p* = 0.999) (Figure 2(b)). These results indicated that the sCD206 level was closely associated with ILD, especially with RP-ILD despite different MSA types.

**Figure 3:**
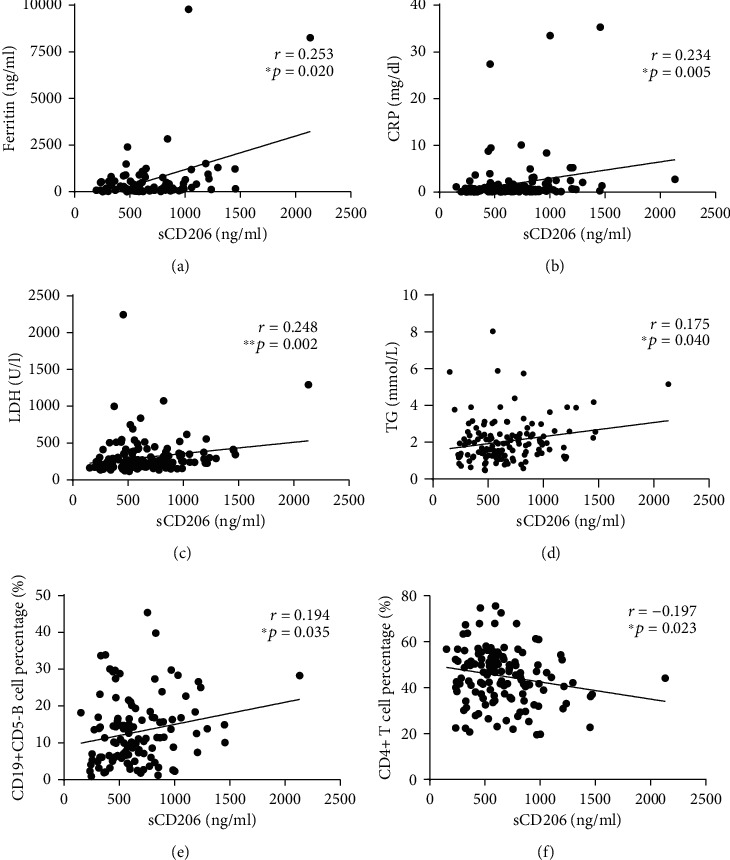
The correlations of sCD206 levels with laboratory parameters in patients with DM. (a) sCD206 levels were positively correlated with ferritin levels in patients with DM. (b) sCD206 levels were positively correlated with CRP levels in patients with DM. (c) sCD206 levels were positively correlated with LDH levels in patients with DM. (d) sCD206 levels were positively correlated with TG levels in patients with DM. (e) sCD206 levels were positively correlated with CD19+CD5- B cell percentage in patients with DM. (f) sCD206 levels were negatively correlated with CD4+T cell percentage in patients with DM. sCD206: soluble CD206; DM: dermatomyositis; CRP: C-reactive protein; LDH: lactate dehydrogenase; TG: triglyceride.

The correlations between sCD206 levels and other clinical features such as muscle weakness/myalgia, skin manifestations (including heliotrope sign, Gottron papules, mechanic's hands, and Raynaud phenomenon), arthralgia, dysphagia, and internal malignancy were also analysed. However, no significant difference in the sCD206 level was found between patients with these clinical features and those without them (*p* values all greater than 0.05, data not shown). In addition, sCD206 levels showed no difference between patients who were treatment naïve and those who were exposed to treatment (median: 534.3 ng/ml, IQR: 420.7-723.0 ng/ml vs. median: 603.6 ng/ml, IQR: 457.7-823.8 ng/ml, *p* = 0.189). In terms of laboratory examinations, the concentrations of sCD206 were found to be positively correlated with levels of serum ferritin (*r* = 0.253, *p* = 0.020), C-reactive protein (CRP) (*r* = 0.234, *p* = 0.005), lactate dehydrogenase (LDH) (*r* = 0.248, *p* = 0.002), triglyceride (TG) (*r* = 0.175, *p* = 0.040), and CD19+CD5- B cell percentage (*r* = 0.194, *p* = 0.035), while they were negatively correlated with the CD4+ T cell percentage (*r* = −0.197, *p* = 0.023) (Figures 3(a)–3(f)). No association was found between the sCD206 level and the creatine kinase (CK) level, erythrocyte sedimentation rate (ESR), immunoglobulin (IgG) level, CD3+ T cell percentage, CD8+ T cell percentage, or CD19+CD5+ B cell percentage (*p* values all greater than 0.05).

### 3.4. Correlations between sCD206 Levels and Pulmonary Function Test Parameters in Patients with DM

**Figure 4:**
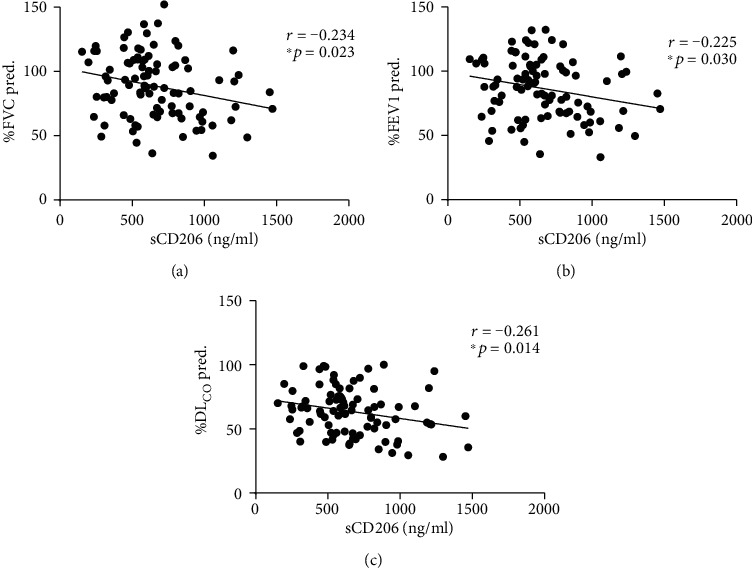
The correlation between sCD206 levels and PFTs in patients with DM. (a) sCD206 levels were negatively correlated with %FVC in patients with DM. (b) sCD206 levels were negatively correlated with %FEV1 in patients with DM. (c) sCD206 levels were negatively correlated with %DL_CO_ in patients with DM. sCD206: soluble CD206; DM: dermatomyositis; PFTs: pulmonary function tests; %FVC: percent predicted forced vital capacity; %FEV1: percent predicted forced expiratory volume in one second; %DL_CO_: percent predicted carbon monoxide diffusion capacity.

To test the association of sCD206 with the severity of lung involvement, we also analysed the correlations between the sCD206 levels and pulmonary function test (PFT) data, including percent predicted forced vital capacity (%FVC), percent predicted forced expiratory volume in one second (%FEV_1_), and percent predicted carbon monoxide diffusion capacity (%DL_CO_). The results showed that all these three parameters were negatively correlated with the sCD206 concentrations (*r* = −0.234, -0.225, and -0.261, and *p* = 0.023, 0.030, and 0.014, respectively) (Figure 4). The results suggested that DM patients with higher serum sCD206 levels tended to have more severe pulmonary involvement.

### 3.5. Predictive Values of sCD206 in DM Patients with RP-ILD

**Figure 5:**
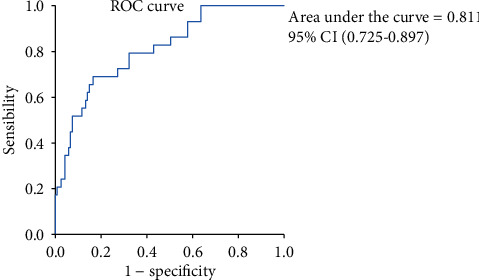
ROC curve for the RP-ILD risk prediction model. ROC curve analysis was used to assess the diagnostic value of sCD206 levels in the prediction of RP-ILD. The area under the curve was 0.811 (*p* < 0.0001), and the optional cut-off value was 792.75 ng/ml with a sensibility of 0.690 and a specificity of 0.835. ROC: receiver operating characteristic; RP-ILD: rapidly progressive interstitial lung disease; sCD206: soluble CD206.

As previously shown in the results, sCD206 was closely associated with RP-ILD; then, we further calculated the optimal cut-off value for sCD206 to predict RP-ILD by using a ROC curve. The AUC was 0.811 (95% CI: 0.725-0.897, *p* < 0.0001), and the optimal cut-off value was 792.75 ng/ml, with a sensitivity of 0.690 and a specificity of 0.835 (Figure 5). Furthermore, the positive predictive value (PPV) and negative predictive values were 50% and 92%, respectively. Similarly, we calculated the optimal cut-off values for ferritin (417.7 ng/ml) and %FVC (72.65%) to predict RP-ILD as well (with limited cases). The AUCs for ferritin and %FVC were 0.798 and 0.859, respectively. As shown in Figure 5, the AUC for sCD206 in RP-ILD was 0.811, close to that of serum ferritin, suggesting comparable values of sCD206 and ferritin in predicting DM-associated RP-ILD.

Then, univariate as well as age- and gender-adjusted multivariate analyses were conducted to reveal factors associated with high risk of RP-ILD in DM patients, and detailed results are shown in Table 2. Among the biomarkers, anti-MDA-5 antibody, sCD206, ferritin, and ESR were associated with RP-ILD. As regards skin involvement, we found that mechanic's hands but not skin ulcer was associated with RP-ILD. In the age- and gender-adjusted multivariate logistic regression analysis, those statistically significant differences in the biomarkers mentioned above still existed, suggesting that these predictive factors were independent risk factors for RP-ILD.

### 3.6. Correlation between sCD206 Levels and Disease Activities

During the longitudinal study, sera from 20 patients who visited the hospital more than twice were obtained to determine sCD206 levels. During every follow-up visit, disease activities were assessed with a follow-up duration of 4.8 (3.0-7.0) months. The basic clinical characteristics of the 20 patients in longitudinal follow-up are displayed in Table 3.

**Figure 6:**
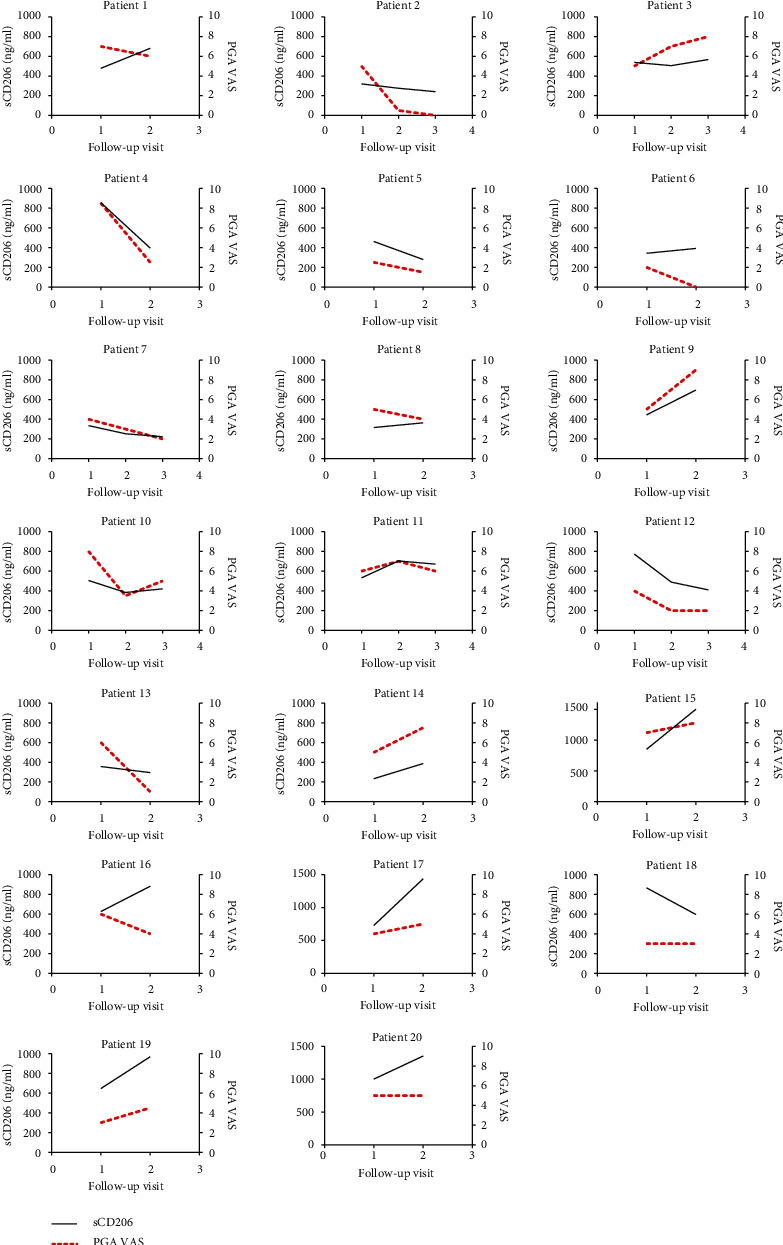
The longitudinal analysis of sCD206 levels and PGA VAS in 20 patients with DM, respectively. sCD20: soluble CD206; DM: dermatomyositis; VAS: visual analog scale; PGA: physician global assessment.

There was a positive correlation between the sCD206 level and the PGA VAS of DM patients by GEE analysis, with a statistically significant difference (*β* = 54.201, *p* = 0.001). The sCD206 levels and PGA VAS in 20 patients with DM at each visit are shown in Figure 6.

## 4. Discussion

The study showed that the serum levels of sCD206 were significantly elevated in patients with DM, especially in cases where DM was complicated with RP-ILD, and there were no correlations between sCD206 levels and MSA types in patients with RP-ILD. Furthermore, an elevated sCD206 level was a risk factor for RP-ILD in DM patients. In addition, we also conducted a follow-up study in some of the patients, and the results showed that the change in the serum sCD206 level was positively correlated with PGA VAS, suggesting that the level of sCD206 could be used to monitor disease activity.

Horiike et al. confirmed the elevation of serum sCD206 level in MDA-5-positive DM-ILD patients and found that the increase was more evident in nonsurvivor patients, suggesting sCD206 as a serum biomarker for predicting the severity of MDA-5-DM-ILD patients [29]. Our study divided patients with ILD into RP-ILD and nonRP-ILD groups, and the results showed that sCD206 levels were significantly elevated in RP-ILD patients, while there was no significant difference between patients with nonRP-ILD and patients without ILD. It can be noted from the result that sCD206 level was more closely associated with RP-ILD rather than ILD. Furthermore, there was no difference in sCD206 levels in RP-ILD patients bearing different MSA types, suggesting that the marked elevation of sCD206 was not specific for patients with anti-MDA-5 autoantibodies but also those with other MSA types. In addition, age- and gender-adjusted multivariate analysis indicated that sCD206 was an important early warning indicator for RP-ILD in DM patients.

To date, multiple predictors of RP-ILD have been published in several studies. In addition to the anti-MDA-5 antibody and ferritin, which were already known to be associated with RP-ILD, ESR, CRP, lymphocyte, and T cell counts can also be used as predictive biomarkers for RP-ILD [7, 37–39]. In our study, the levels of sCD206, as well as anti-MDA-5 antibody, ferritin, and ESR, were correlated with RP-ILD. Furthermore, sCD206 and ferritin had a comparable predictive value for RP-ILD with moderate diagnostic value, suggesting that sCD206 could also be used for the prediction of RP-ILD in clinical practice.

It has been reported that macrophage activation is critically involved in the development of ILD in patients with DM [40–42] and that CD206 is an indicator of macrophage activation [23, 27]. The expression of CD206 was significantly increased in the lung tissues of patients with idiopathic pulmonary fibrosis [43], suggesting that CD206 might participate in the pathogenesis of pulmonary fibrosis. However, CD206 may not be specific for DM, as the elevation of sCD206 was also found in other diseases with macrophage activation, including sepsis and liver disease [28]. Interestingly, high levels of CD206 were also found in several connective diseases such as systemic lupus erythematosus and rheumatoid arthritis [26, 44–46]. Despite this, our study provided additional evidence supporting that macrophage activation may contribute to the pathogenesis of DM-associated ILD; however, the exact role CD206 played needs to be further clarified.

There were several limitations of this study. First, the sample size was relatively small, and the number of patients in each group after classification into MSA subtypes was small, thus preventing further analysis. Second, MSAs were only detected by immunoblot, which may lead to potential false-positive and false-negative results. In addition, in this particular study, assessment of outcome measures as related to sCD206 levels was not conducted. Further studies with larger sample size are needed to further verify the associations between sCD206 and RP-ILD as well as patients' prognosis.

## 5. Conclusions

The serum level of sCD206 was closely associated with ILD, especially with RP-ILD in DM patients with all types of MSA; thus, sCD206 can be used as a serum biological predictor of RP-ILD in patients with DM. The concentrations of sCD206 could reflect the disease activities during patients' disease course. Further studies are needed to explore the role of sCD206 in the pathogenesis of DM with RP-ILD.

## Figures and Tables

**Table 1:** Clinical characteristics of patients with DM.

Characteristics	Patients with DM % (*n*/*N*) or mean ± SD/median (IQR)
Female/male ratio	108/42
Onset age (yrs, mean ± SD)	48.87 ± 13.05
Disease duration (months, median (IQR))	8.5 (2.4-24)
Treatment naïve	32% (48/150)
Clinical features	
Muscle weakness	66.7% (100/150)
Myalgia	39.3% (59/150)
Heliotrope sign	50.7% (76/150)
Gottron papules	58.7% (88/150)
Mechanic's hands	33.3% (50/150)
Raynaud phenomenon	7.3% (11/150)
Skin ulcer	19.3% (29/150)
Arthritis/arthralgia	32% (48/150)
Dysphagia	27.3% (41/150)
ILD	68% (102/150)
RP-ILD	19.3% (29/150)
Malignancy	15.3% (23/150)
Laboratory examinations	
ANA-positive	32.9% (47/143)
Anti-ARS	20.7% (31/150)
Anti-MDA-5	26% (39/150)
Anti-TIF1*γ*	13.3% (20/150)
Anti-NXP2	5.3% (8/150)
Anti-Mi-2	5.3% (8/150)
Anti-SAE	2% (3/150)
MSA negative	27.3% (41/150)
CK levels (26-200 IU/l)	80 (37-253)
LDH levels (100-250 IU/l)^a^	237 (183-342.5)
CRP levels (<0.8 mg/dl)^b^	0.518 (0.187-1.158)
ESR levels (<20 mm/H)^c^	15 (7-38.25)
Ferritin levels (11-306.8 ng/ml)^d^	208.8 (87.65-604.9)
IgG (694-1620 mg/dl)^e^	1210 (1003-1480)
CD3+ T cell percentage (50.7-86%)^f^	70.7 (64.93-80.13)
CD4+ T cell percentage (23.3-50.2%)^f^	46.45 (37.15-52.55)
CD8+ T cell percentage (12.5-36.9%)^f^	23.85 (16.58-31.33)
CD19+CD5+ B cell percentage (0-0.96%)^g^	3.05 (0.955-7.818)
CD19+CD5- B cell percentage (1.45-9.49%)^g^	11.13 (6.088-16.8)
Pulmonary function tests	
% FVC (%)^h^	88.53 ± 25.06
% FEV_1_ (%)^h^	86.48 ± 23.19
% DL_CO_ (%)^i^	63.53 ± 18.25

DM: dermatomyositis; ILD: interstitial lung disease; ANA: anti-nuclear autoantibodies; ARS: aminoacyl-tRNA synthetases; MDA-5: melanoma differentiation-associated gene-5; TIF1*γ*: transcription intermediary factor 1*γ*; NXP-2: nuclear matrix protein-2; Mi-2: nucleosome remodelling deacetylase complex; SAE: small ubiquitin-like modifier-1 activating enzyme; CK: creatine kinase; LDH: lactic dehydrogenase; CRP: C-reactive protein; ESR: erythrocyte sedimentation rate; IgG: immunoglobulin; %FVC: percent predicted forced vital capacity; %FEV_1_: percent predicted forced expiratory volume in one second; %DL_CO_: percent predicted carbon monoxide diffusion capacity. ^a,b,c,d,e,f,g,h,i^Data were available for 149, 140, 142, 85, 144, 134, 118, 94, and 89 patients, respectively.

**Table 2:** Predictive factors for RP-ILD in DM patients using univariate and age- and gender-adjusted multivariate logistic regression analyses.

Predictor	Univariate analysis			Age- and gender-adjusted multivariate analysis		
	Odds ratio	95% CI	*p* value	Odds ratio	95% CI	*p* value
Age (yrs)	1.005	0.974-1.036	0.767			
Gender (female)	1.279	0.501-3.264	0.607			
Anti-MDA-5-positive	3.584	1.530-8.393	0.003	3.910	1.629-9.382	0.002
Anti-ARS-positive	2.025	0.813-5.042	0.130	1.988	0.795-4.974	0.142
sCD206 (ng/ml)	1.004	1.003-1.006	<0.0001	1.005	1.003-1.007	<0.0001
CRP (mg/dl)^a^	1.054	0.981-1.131	0.149	1.052	0.979-1.131	0.166
ESR (mm/H)^b^	1.019	1.003-1.035	0.017	1.020	1.004-1.036	0.015
Ferritin (ng/ml)^c^	1.002	1.001-1.003	0.002	1.002	1.001-1.004	0.002
%FVC (%)^d^	0.929	0.893-0.966	<0.0001	0.928	0.892-0.964	<0.0001
CK (IU/l)	1.000	1.000-1.000	0.989	1.000	1.000-1.000	0.964
Muscle weakness	0.542	0.237-1.240	0.147	0.541	0.235-1.245	0.149
Heliotrope sign	0.748	0.331-1.688	0.485	0.752	0.333-1.699	0.493
Gottron papules	1.732	0.729-4.113	0.213	1.734	0.729-4.123	0.213
Mechanic's hands	2.633	1.151-6.023	0.022	2.593	1.125-5.976	0.025
Skin ulcer	1.432	0.544-3.769	0.467	1.517	0.567-4.059	0.406
Arthritis/arthralgia	1.150	0.488-2.707	0.750	1.143	0.485-2.695	0.760
Dysphagia	0.641	0.240-1.709	0.374	0.613	0.224-1.675	0.340
Elevated sCD206 (≥792.75 ng/ml)	11.222	4.466-28.200	<0.0001	12.679	4.844-33.182	<0.0001
Elevated ferritin (≥417.7 ng/ml)^c^	7.837	2.690-22.830	<0.0001	11.416	3.383-38.525	<0.0001

DM: dermatomyositis; RP-ILD: rapidly progressive interstitial lung disease; sCD206: soluble CD206; ARS: aminoacyl-tRNA synthetases; MDA-5: melanoma differentiation-associated gene-5; CRP: C-reactive protein; ESR: erythrocyte sedimentation rate; CK: creatine kinase; %FVC: percent predicted forced vital capacity. ^a,b,c,d^Data were available for 140, 142, 85, and 94 patients, respectively.

**Table 3:** Basic clinical characteristics of DM patients with follow-up data.

	Gender (F/M)	Onset age/years	Disease course/months	Follow-up duration/months	Treatment naïve	ILD types	PGA VAS at enrollment
Patient 1	F	45	4	0.80	Yes	Without	7
Patient 2	M	46	4	30.97	Yes	Without	5
Patient 3	M	36	7	11.70	Yes	NonRP-ILD	5
Patient 4	F	37	4	7.03	No	RP-ILD	8.5
Patient 5	F	60	2	5.63	No	NonRP-ILD	2.5
Patient 6	M	27	1	5.03	No	NonRP-ILD	2
Patient 7	F	49	24	11.80	No	NonRP-ILD	4
Patient 8	F	40	2	3.47	Yes	NonRP-ILD	5
Patient 9	F	50	1	4.50	Yes	NonRP-ILD	5
Patient 10	F	59	24	5.67	No	RP-ILD	8
Patient 11	M	45	3	2.03	No	RP-ILD	6
Patient 12	F	43	36	13.33	No	NonRP-ILD	4
Patient 13	M	36	2	4.03	Yes	Without	6
Patient 14	M	39	7	6.97	No	NonRP-ILD	5
Patient 15	F	38	36	3.50	No	NonRP-ILD	7
Patient 16	M	69	9	2.83	Yes	NonRP-ILD	6
Patient 17	F	83	1	2.47	Yes	Without	4
Patient 18	F	47	5	6.17	No	NonRP-ILD	3
Patient 19	M	60	2	2.57	No	RP-ILD	3
Patient 20	F	64	29	3.50	No	NonRP-ILD	5

DM: dermatomyositis; ILD: interstitial lung disease; RP-ILD: rapidly progressive interstitial lung disease; VAS: visual analog scale; PGA: physician global assessment.

## Data Availability

All data used to support the findings of this study are available from the corresponding authors upon request.
